# Intermolecular Interactions in G Protein-Coupled Receptor Allosteric Sites at the Membrane Interface from Molecular Dynamics Simulations and Quantum Chemical Calculations

**DOI:** 10.1021/acs.jcim.2c00788

**Published:** 2022-09-30

**Authors:** Tianyi Ding, Dmitry S. Karlov, Almudena Pino-Angeles, Irina G. Tikhonova

**Affiliations:** School of Pharmacy, Medical Biology Centre, Queen’s University Belfast, Belfast, Northern IrelandBT9 7BL, U.K.

## Abstract

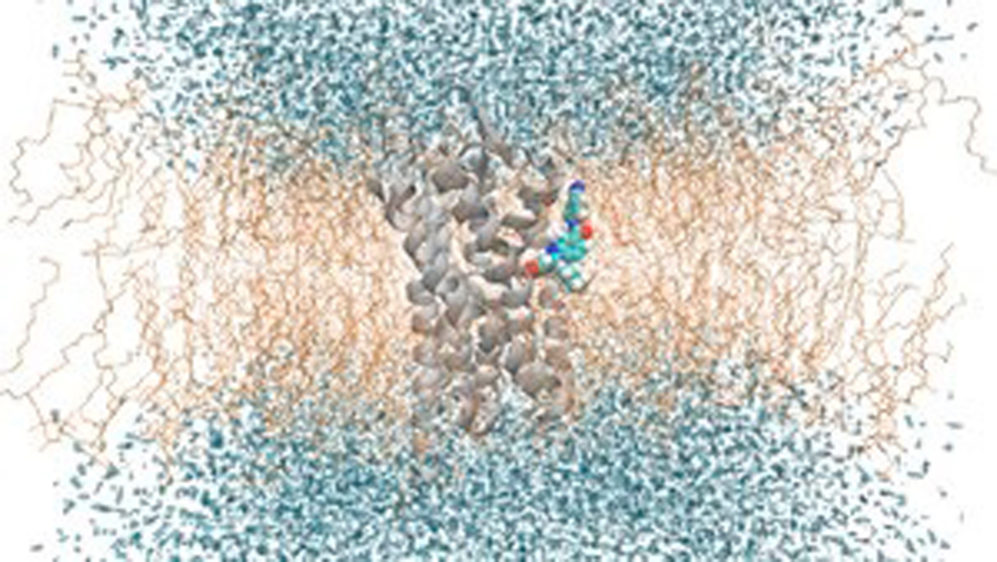

Allosteric modulators are called promising candidates in G protein-coupled receptor (GPCR) drug development by displaying subtype selectivity and more specific receptor modulation. Among the allosteric sites known to date, cavities at the receptor–lipid interface represent an uncharacteristic binding location that raises many questions about the ligand interactions and stability, the binding site structure, and how all of these are affected by lipid molecules. In this work, we analyze interactions in the allosteric sites of the PAR2, C5aR1, and GCGR receptors in three lipid compositions using molecular dynamics simulations. In addition, we performed quantum chemical calculations involving the symmetry-adapted perturbation theory (SAPT) and the natural population analysis to quantify the strength of intermolecular interactions. We show that besides classical hydrogen bonds, weak polar interactions such as O–HC, O–Br, and long-range electrostatics with the backbone amides contribute to the stability of allosteric modulators at the receptor–lipid interface. The allosteric cavities are detectable in various membrane compositions. The availability of polar atoms for interactions in such cavities can be assessed by water molecules from simulations. Although ligand–lipid interactions are weak, lipid tails play a role in ligand binding pose stability and the size of allosteric cavities. We discuss physicochemical aspects of ligand binding at the receptor–lipid interface and suggest a compound library enriched by weak donor groups for ligand search in such sites.

## Introduction

G protein-coupled receptors (GPCRs) are cell surface receptors comprised of seven transmembrane (TM)-spanning helices that are capable of binding to a variety of endogenous molecules outside of the cell to activate a complex chain of biological events inside the cell. Receptor functional states can be modulated by small molecules or peptides acting at allosteric sites that are topographically distinct from sites of endogenous molecules. The allosteric modulators (AMs) can change the affinity and/or efficacy of endogenous molecules or other orthosteric ligands such as agonists and antagonists, as well as GPCRs’ constitutive activity. Thus, positive allosteric modulators (PAMs) increase GPCR signal transduction, whereas negative allosteric modulators (NAMs) decrease it. Furthermore, there are also neutral AMs that block the activity of PAMs and NAMs but do not affect the response to the orthosteric agonist. AMs have many potential benefits as medicines such as better control of on-target dose-related side effects, reduced off-target effects, and pathway selectivity.^1−3^ Until recently, the discovery of allosteric modulators was mainly achieved via high-throughput screening. However, recent achievements in crystallography and cryoelectron microscopy (cryo-EM) have disclosed the crystal structures of several GPCRs bound to AMs, providing opportunities for structure-based allosteric drug discovery.^4,5^

The experimental structures of GPCR–AM complexes show that AMs can bind inside and outside of a GPCR helical core. Thus, in the crystal structures of the M_2_ muscarinic and CCR5 chemokine receptors, AMs are found inside the receptor core on the extracellular side.^6,7^ In contrast, AMs of CCR2, CCR7, CCR9, and the β_2_ adrenergic receptors sit within the intracellular side of the receptor core.^8−11^ AMs also are found to bind outside of the receptor helical core at the various locations of the lipid interface. Thus, AMs bind at the lipid interface of helices 1–3 in the glucagon-like peptide-1 (GLP-1R) and the P_2_Y_1_ purinergic receptors;^12,13^ helices 2–4 in the cannabinoid 1 (CB_1_) and protease-activated 2 (PAR_2_) receptors;^14,15^ helices 3–4 in the free fatty acid receptor one (FFA_1_);^16,17^ helices 3–5 and second intracellular loop 2 (IL2) in FFA_1_, the β_2_ adrenergic, the C5a anaphylatoxin chemotactic 1 (C5aR1), and the dopamine D_1_ receptors;^16,18−21^ helices 6–7 in the GLP-1R and the glucagon receptor (GCGR);^22,23^ and helices 6-7-1 in the adenosine receptor (A1).^24^ Hedderich et al.^25^ have recently docked small molecular probes to 557 GPCR structures and predicted new previously uncharacterized allosteric sites. In addition, the detailed analysis of the available experimental receptor–AM complexes at the lipid interface in two reviews^26,27^ shows that a significant surface area of AMs is exposed to the lipid bilayer (from ∼20 to ∼50%), highlighting the importance of a membrane environment for ligand binding. The structural data provide a first detailed static picture of GPCR–AM interactions. However, little information is known on the dynamics of these interactions in the physiological condition, which is a key aspect to fully understanding receptor regulation by allosteric sites for future drug design efforts.

Molecular dynamics (MD) simulation is a suitable computational tool for studying the dynamics of GPCRs in a membrane environment. The MD simulations were performed to study the binding and interactions of ligands in allosteric sites in several GPCR crystal complexes. Thus, allosteric communication between the PAM LY2119620 and the agonist iperoxo linked to rotameric changes of aromatic residues was proposed from the MD simulations of the M_2_ receptor complexes.^28^ In other studies, the conventional and enhanced sampling simulations of the P2Y1 receptor with the NAM BPTU showed a critical hydrogen bond holding the ligand in the site and the contribution of the lipid bilayer in the ligand recognition process.^29,30^ The binding interactions, pathways, and cooperation of the PAM AP8 and MK-8666 at FFA1 were explored in the conventional and supervised MD simulations.^31,32^ We have recently combined enhanced sampling MD simulations with a fragment-based approach to map and characterize GPCR allosteric cavities at different locations.^33^

In this study, we focus on allosteric binding sites at the receptor–lipid interface to further delineate the interactions within the sites. We study three receptors, i.e., PAR2, C5aR1, and GCGR, whose AM binds at different locations of the receptor–lipid interface. Although the importance of these peptide GPCRs has been shown in inflammation (PAR2 and C5aR1)^34,35^ and diabetes (GCGR),^36^ targeting these receptors by small-molecule ligands via the orthosteric site has been challenging, thus, stimulating exploration of other sites. We perform multiple MD simulations of the receptors in AM-bound and empty forms, totaling 26.25 μs, to rigorously characterize AM interactions in allosteric binding cavities that occur as a result of interactions with three different lipid compositions. We choose 1-palmitoyl-2-oleoyl-*sn*-glycero-3-phosphocholine (POPC), 1,2-dimyristoyl-*sn*-glycero-3-phosphocholine (DMPC), and POPC with a 10% concentration of cholesterol (POPC-Chol) as lipid bilayers. Phosphatidylcholines are selected as they represent the most abundant lipids in the animal cell membrane.^37^ POPC and DMPC are distinct in the length and degree of saturation, which affect membrane ordering and melting temperature. Cholesterol decreases membrane order below melting temperature and participates in lipid raft formation when the membrane melts. We, therefore, wanted to see how the change in membrane stiffness affects ligand binding at the protein–lipid interface. The importance of lipids and cholesterol in GPCRs functional dynamics has been shown in numerous studies.^38−41^ Our results demonstrate the key dynamic interactions of AMs, the properties of allosteric cavities, and the contribution of the membrane. Finally, we complement our MD simulations with quantum chemical calculations to further evaluate the energetic contribution of a residue sidechain and backbone in the ligand–receptor interactions using the symmetry-adapted perturbation theory (SAPT).^42^ One of the SAPT method realizations, F/I-SAPT,^43,44^ provides the decomposition of the pairwise interaction energy of selected fragments of two molecules into electrostatic, exchange repulsion, polarization, and dispersion components, helping to delineate the nature and strength of interactions. Next, the wave function from quantum chemical calculations was transformed to localized forms of one- and two-center orbitals (core, lone pairs, and covalent bonds) using the natural population analysis.^45,46^ Computation of charge transfer, *Q*_CT_, from a donor orbital to an acceptor orbital provides a descriptor evaluating the strength of an atom–atom interaction.^47,48^*Q*_CT_ of polar contacts in the ligand–receptor complexes was compared with *Q*_CT_ of the classical and nonclassical hydrogen bonds with the optimal geometry.

Together, our study shows that in addition to classical hydrogen bonds such as O/N–HO/N bonds, weak polar interactions such as nonclassical hydrogen bonds, O–HC, halogen bonds, O–Br, and long-range electrostatics could be important when targeting a site at the receptor–lipid interface. Such weak polar interactions become favorable in the lipid environment as hydrophobic lipid tails cannot saturate and screen these interactions and, therefore, can be considered in the design and optimization of small-molecule ligands binding at the protein–lipid interface.

## Results

### PAR2 in Complex with AZ3451

**Figure 1:**
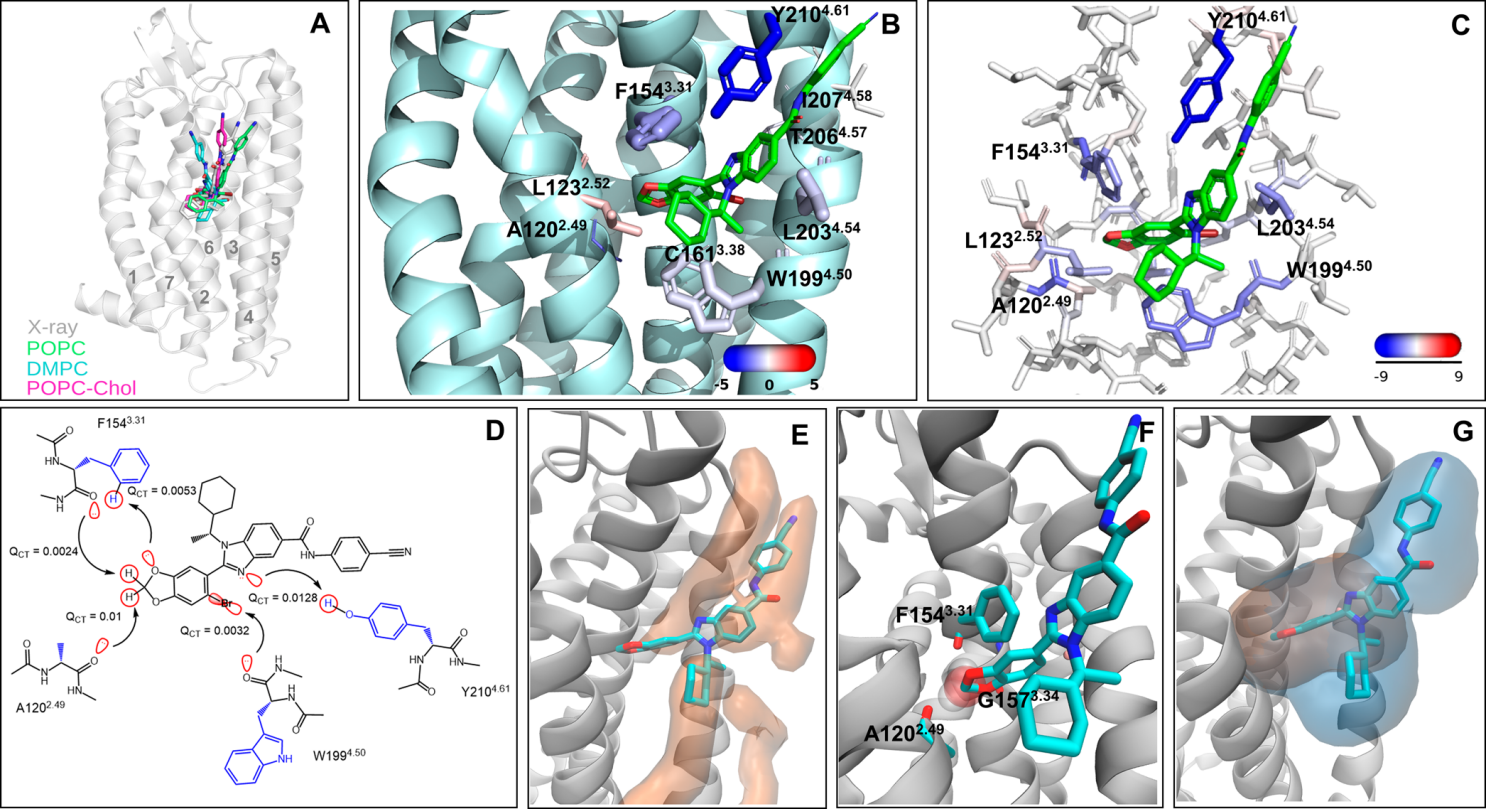
Extrahelical allosteric site of PAR2 and its interactions from MD simulations and quantum chemical calculations. (A) Overall location of AZ3451 in PAR2. The overlay of the X-ray position with the average position of AZ3451 in the POPC, DMPC, and POPC-Chol simulations. The helices are labeled. (B) Zoomed view of the binding site. The key residues forming contacts with AZ3451 are shown in stick representation. The size and color of the residues correspond to the relative strength of van der Waals (vdW) and electrostatic interactions with the ligand, respectively. The actual values of the interaction energies are shown in Table S1. (C) Electrostatic energy from the F/I-SAPT calculations mapped to the allosteric site residues. (D) Two-dimensional (2D) view of the key AZ3451–PAR2 interactions and the value of the *Q*_CT_ descriptor. The backbone and the side chain of residues are colored black and blue. The orbital interactions responsible for charge transfer (*Q*_CT_) between a donor and an acceptor are visualized in red. The direction of the charge transfer is shown by arrows. The backbone and the side chain of residues are colored black and blue. (E) Low-energy lipid area (in orange surface) in the allosteric site obtained from the grid free energy (GFE) calculation based on the POPC simulations of the receptor empty form. The ligand is shown for clarity of the allosteric site location. (F) Overlay of AZ3451 (in a stick) and a water molecule (in transparent SPK representation) from the MD simulations. In the simulations of the receptor empty form, a water molecule frequently occupies the binding pocket of the dioxolane moiety forming H-bonding interactions with the backbone of A120, F154, and G157. (G) Overlay of the allosteric cavities from MDpocket calculation with the selection of only receptor atoms (orange surface) and receptor–lipid atoms (blue surface). The ligand is shown for clarity of the binding site location. The results are shown for the POPC simulations, and the others can be found in Figure S2. The lipid atoms were selected at a distance of 6 Å from the selected receptor atoms.

The allosteric ligand AZ3451 (*K*_d_ = 13.5 nM) is located outside of the transmembrane helical bundle in a pocket created by helices 2, 3, and 4^15^ (Figure 1A,B). In our MD simulations, the receptor–ligand complex is stable in all membrane compositions with a low average root-mean-square deviation (RMSD) for the protein backbone and ligand nonhydrogen atoms (Table 1). The ligand has higher mobility in the DMPC membrane compared to the POPC and POPC-Chol membranes, as shown by the root-mean-square fluctuation (RMSF) (Table 1). The benzonitrile moiety is the most dynamic part of the ligand in all of the simulations.

**Table 1:** Root-Mean-Square Deviation (RMSD) and Fluctuation (RMSF) of PAR2, C5aR1, and GCGR and Their Allosteric Ligand in Different Lipid Compositions^a^

systems	RMSD protein, Å	RMSF protein, Å	RMSD ligand, Å	RMSF ligand, Å	RMSF of the most ligand dynamic fragment, Å
**PAR2**					
POPC-L	1.6 ± 0.3	1.1 ± 0.2	1.6 ± 0.3	1.1 ± 0.2	1.5 ± 0.8
POPC-E	1.6 ± 0.3	1.0 ± 0.2			
DMPC-L	1.9 ± 0.5	1.1 ± 0.2	2.3 ± 0.8	1.5 ± 0.5	2.4 ± 0.8
DMPC-E	2.0 ± 0.5	1.2 ± 0.2			
POPC_Chol-L	1.7 ± 0.4	1.1 ± 0.2	1.8 ± 0.3	1.3 ± 0.3	2.0 ± 0.6
POPC_Chol-E	1.8 ± 0.4	1.2 ± 0.2			
**C5aR1**					
POPC_5O9H-L	1.8 ± 0.4	1.1 ± 0.2	2.5 ± 1.0	1.7 ± 0.7	2.8 ± 1.5
POPC_5O9H-E	1.9 ± 0.5	1.2 ± 0.2			
DMPC_5O9H-L	1.8 ± 0.4	1.2 ± 0.2	4.7 ± 1.6	2.3 ± 0.9	1.8 ± 0.6
DMPC_5O9H-E	1.9 ± 0.4	1.2 ± 0.2			
POPC_Chol_5O9H-L	1.9 ± 0.5	1.1 ± 0.3	2.3 ± 0.9	1.9 ± 0.8	2.6 ± 1.3
POPC_Chol_5O9H-E	1.9 ± 0.5	1.2 ± 0.3			
POPC_6C1Q-L	2.2 ± 0.6	1.5 ± 0.3	1.3 ± 0.3	0.8 ± 0.3	0.9 ± 0.5
DMPC_6C1Q-L	1.7 ± 0.4	1.2 ± 0.3	0.8 ± 0.3	0.7 ± 0.2	0.7 ± 0.5
POPC_Chol_6C1Q-L	1.5 ± 0.3	1.0 ± 0.2	1.0 ± 0.4	0.8 ± 0.4	0.9 ± 0.7
**GCGR**					
POPC-L	2.4 ± 0.6	1.3 ± 0.4	1.5 ± 0.5	1.2 ± 0.3	1.3 ± 0.5
POPC-E	2.8 ± 0.9	1.6 ± 0.4			
DMPC-L	2.1 ± 0.5	1.2 ± 0.3	1.3 ± 0.3	1.1 ± 0.3	1.2 ± 0.5
DMPC-E	2.2 ± 0.5	1.3 ± 0.3			
POPC_Chol-L	2.3 ± 0.6	1.3 ± 0.3	1.3 ± 0.4	1.0 ± 0.3	1.2 ± 0.5
POPC_Chol-E	2.8 ± 1.0	1.6 ± 0.4			

a RMSD and RMSF values are calculated based on the Cα atoms and nonhydrogen atoms for the receptor and ligand, respectively. The RMSD and RMSF calculations were performed for the ligand-bound (-L) and empty (-E) forms of the receptors. The RMSF of the most dynamic ligand fragment, involving benzonitrile of AZ3451 in PAR2, *N*-(1,3-benzodioxol-5-ylmethyl)ethanamine of NDT9513727 in C5aR1, and 1,3-dichlorobenzene of MK-0893 in GCGR is shown.

From the ligand–residue pairwise average interaction energy (Figure 1B and Table S1), AZ3451 forms electrostatic interactions with Y210^4.61^ (the Ballesteros–Weinstein numbering scheme in the superscript^49^), A120^2.49^, and F154^3.31^, with electrostatic energies of −5.1, −2.8, and −1.8 kcal/mol, respectively. Among them, the hydroxyl group of Y210^4.61^ only forms a hydrogen bond with the ligand through the nitrogen atom of the benzimidazole (Figure 1B). This hydrogen bond is formed throughout the simulation time, with an average occupancy of ∼65% in the POPC and POPC-Chol membranes and 53% in the DMPC membrane at a 3.2 Å distance cutoff (Table S2). Interestingly, mutation of Y210^4.61^ to leucine leads to a 25-fold decrease in the activity and not to the absence of binding, suggesting that other interactions contribute to ligand binding.^15^ The electrostatic interactions with A120^2.49^ and F154^3.31^ are expected to be through the backbone of these residues and the nearby benzodioxole of AZ3451 (Figure 1B). Besides the polar interactions, AZ3451 forms van der Waals (vdW) interactions with F154^3.31^, L203^4.54^, W199^4.50^, Y210^4.61^, and L123^2.52^ with a vdW energy below −3 kcal/mol. From the calculation of the average ligand–lipid interaction energy along the simulated trajectories (Table S1), we saw that AZ3451 is engaged in vdW interactions with four lipid tails in all of the membrane compositions, and the benzonitrile moiety of the ligand forms a polar interaction with a lipid head group in POPC and DMPC.

To further quantify AZ3451–PAR2 interactions, we conducted F/I-SAPT interaction energy decomposition based on the geometry of the ligand binding site optimized at a DFT level (Table S3). The F/I-SAPT interaction energy was calculated between the ligand and a residue fragment, involving either a residue backbone amide or a side chain. The strongest interaction is between the ligand and the side chain of Y210^4.61^, involving the relatively similar contributions of electrostatics and dispersion components, −8.58 and −10.35 kcal/mol, respectively (Table S3). We then separately computed SAPT interaction energy of the Y210^4.61^ side chain with amidobenzonitrile of AZ3451, which predominantly forms aromatic interactions, and with benzimidazole of AZ3451, which has a hydrogen bond with the ligand (Table S4). The total energy was −4.94 and −7.62 kcal/mol, respectively, indicating a comparable contribution of both the H-bond and aromatic interactions. The reason for the weak hydrogen bond is due to the π–π stacking interaction of Y210^4.61^ and benzimidazole of the ligand, which sterically confines the side chain.

More careful consideration of the potential energy function in the F/I-SAPT calculations highlighted the electrostatic contribution of several residues in the interaction with AZ3451 (Figure 1C). Thus, we saw a significant electrostatic component of the F154^3.31^ and A120^2.49^ backbone amides with energies of −5.5 and −5.37 kcal/mol, respectively, and the W199^4.50^ side chain and backbone amide with energies of −4.3 and −3.72 kcal/mol, respectively (Table S3). In the case of F154^3.31^ and A120^2.49^ amides, the joint induction component, reflecting polarization of the molecules, was about −1.3 kcal/mol, which is only below −3.15 kcal/mol of the Y210^4.61^ side chain. The amides of F154^3.31^ and A120^2.49^ are engaged in polar interactions with benzodioxole of the ligand with a possibility of forming a nonclassical O–HC bond between the carbonyl group of the backbone and the methylene hydrogens of benzodioxole (Figure 1C). While the W199^4.50^ side chain is engaged in aromatic and vdW interactions with the ligand, the W199^4.50^ amide has polar interactions with the nearby Br atom with the possibility of O–Br halogen bond formation. The joint induction component of the W199^4.50^ amide is higher compared to the W199^4.50^ side chain, −0.92 vs −0.33 kcal/mol. Overall, the electrostatic component of the interaction energy with the amide of F154^3.31^, A120^2.49^, and W199^4.50^ is 170% of the Y210^4.61^ side chain electrostatics, demonstrating a significant contribution to the ligand stabilization.

We calculated fragment efficiency as the per-non-hydrogen atom average energy contribution to the F/I-SAPT interaction energy between a residue fragment and the ligand (Table S3). We found higher efficiency of the F154^3.31^ backbone amide compared to the Y210^4.61^ side chain, i.e., −1.97 vs −1.60 kcal/mol. The efficiency of W199^4.50^ and A120^2.49^ amides was also notable, −0.96 and −0.78 kcal/mol, respectively. This supports the importance of the residue amide groups in the stabilization of the ligand.

To evaluate the strength of an atom–atom polar contact, we calculated *Q*_CT_ of the interacting orbitals from the electron density using the natural population analysis. *Q*_CT_ of the polar contact in the ligand–receptor complex was compared with the reference *Q*_CT_ of the polar contacts with the optimized geometry. To obtain the reference *Q*_CT_, we performed a set of relaxed surface scans of fragments involved in classical and nonclassical hydrogen bonds to find the optimal geometry and next the natural population analysis (Figure S1). We found that *Q*_CT_ of a classical hydrogen bond was around 0.05 au for the optimal geometry, while a nonclassical hydrogen bond was 10 times lower.

*Q*_CT_ of ^Y210^O(H–N^sp2^) was 4 times lower than the reference *Q*_CT_ of the optimal H–N hydrogen bond (Figures 1D and S1 and Table S5) and close to the weak nonclassical hydrogen bond, ^A120b^(O–H)C^sp3^, confirming the weakness of this contact. *Q*_CT_ of the O–HC and O–Br bonds (Figure 1D) was close to the reference value of the optimized O–HC and O–Br bonds, indicating the presence of directional contacts.

In the simulations of the receptor empty form, the residues of the allosteric site were not significantly more mobile than in the presence of the ligand, except for Y210^4.61^. The side chain of Y210^4.61^ moved either downward of helix 4 to form a hydrogen bond with L203^4.54^ or T206^4.57^ or upward toward the upper leaflet head groups in all of the membrane systems. The simulations of the empty PAR2 receptor obtained from a PAR2 X-ray structure without AZ3452 (Protein Data Bank (PDB) ID: 5NDD) also showed similar behavior. The average hydrogen bond occupancy for Y210^4.61^ in the empty forms was ∼90% in the POPC and DMPC and ∼63% in the POPC-Chol. The average allosteric site residue–lipid interaction energy showed strong vdW interactions with two lipid tails that occupy the allosteric cavity in the simulations (Table S6). The low-energy lipid area in the allosteric site obtained from the grid free energy calculation based on the POPC simulations of the receptor empty form is shown in Figure 1E. In addition, two other lipid tails and one head group formed interactions with the residues of the allosteric site. In the case of the POPC-Chol membrane, we did not observe cholesterol molecules in the allosteric site within the simulated timeframe. Interestingly, a water molecule occupied the position of the dioxolane moiety of the ligand and interacted with the backbone of A120^2.49^, F154^3.31^, and G157^3.34^ in 51% of the simulation time in the POPC (Figure 1F) and to a less extent in other membrane compositions (Table S7).

We used the MDpocket method^50^ to characterize the properties of the allosteric cavity in the MD trajectories. The allosteric receptor cavity was open and druggable throughout the simulation trajectories of the receptor bound and unbound forms according to the Fpocket 3.0 criteria.^51^ As expected, the calculated receptor cavity volume variation is higher in the empty form compared to the ligand-bound form (Table S8). The volume of the allosteric cavity decreases by ∼59% when only the receptor and not the lipids are used for the volume calculation (Figures 1G and S2). The polar and nonpolar surface area ratio of the allosteric cavity including the protein is 20 and 80%, respectively.

### C5aR1 in Complex with NDT9513727

**Figure 2:**
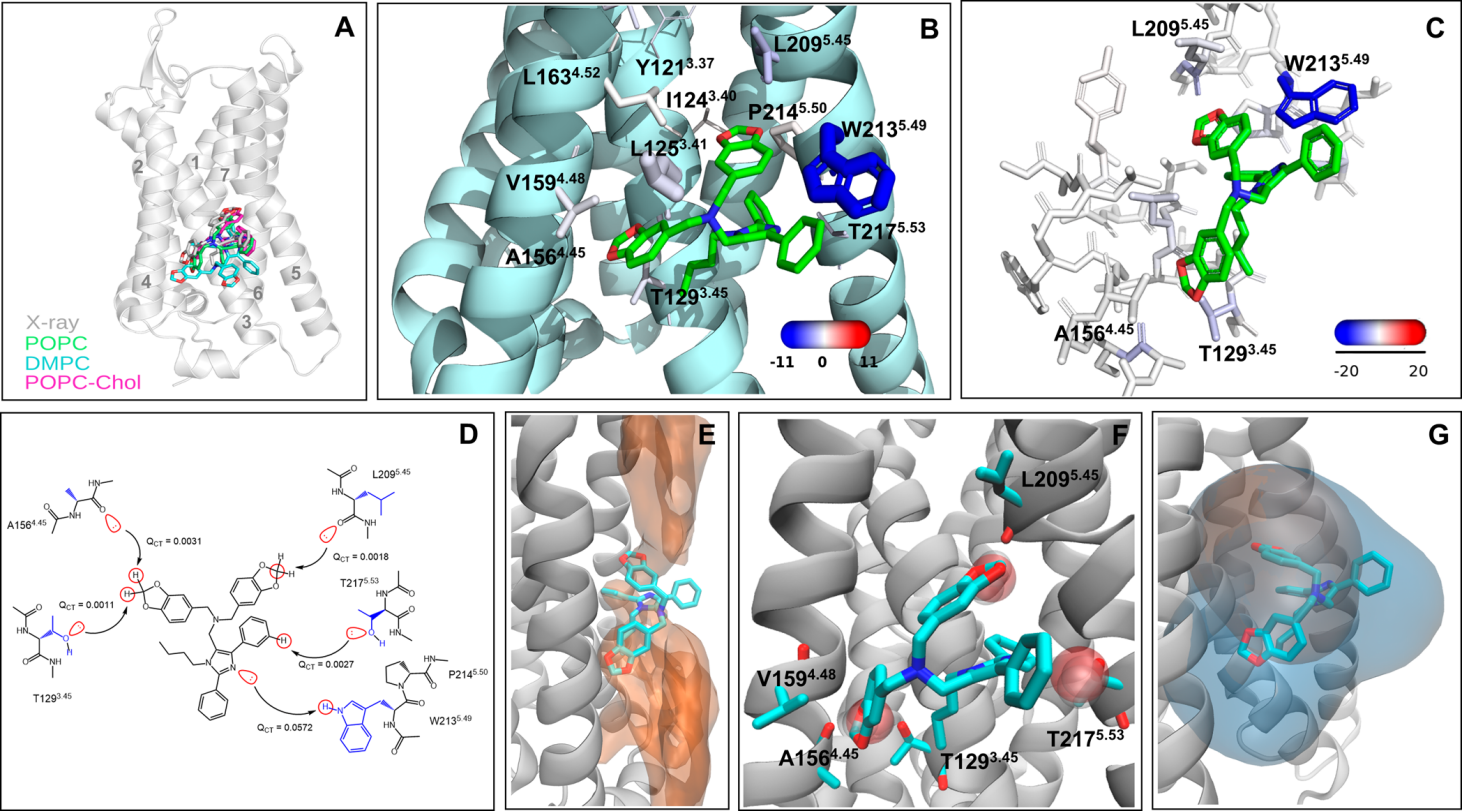
Extrahelical allosteric site of C5aR1 and its interactions from MD simulations and quantum chemical calculations. (A) Overall location of NDT9513727 in C5aR1. The overlay of the X-ray position with the average position of NDT9513727 in the POPC, DMPC, and POPC-Chol simulations. The helices are labeled. (B) Zoomed view of the binding site. The key residues forming contacts with NDT9513727 are shown in stick representation. The size and color of the residues correspond to the relative strength of van der Waals and electrostatic interactions with the ligand, respectively. The actual values of the interaction energies are shown in Table S9. (C) Electrostatic energy from the F/I-SAPT calculations mapped to the allosteric site residues. (D) 2D view of the key NDT9513727–C5aR1 interactions and the value of the *Q*_CT_ descriptor. The orbital interactions responsible for charge transfer (*Q*_CT_) between a donor and an acceptor are visualized in red. The direction of the charge transfer is shown by arrows. The backbone and the side chain of residues are colored black and blue. (E) Low-energy lipid area (in orange surface) in the allosteric site obtained from the grid free energy calculation based on the POPC simulations of the receptor empty form. (F) Overlay of NDT9513727 (in stick) and a water molecule (in transparent SPK representation) from the MD simulations. In the simulations of the receptor empty form, water molecules frequently occupy the binding pocket of the dioxolane ring, forming H-bonding interactions with the backbone of A156, V159, and L209 and the side chain of T129 and T217. (G) Overlay of the allosteric cavities from MDpocket calculation with the selection of only receptor atoms (orange surface) and receptor–lipid atoms (blue surface). The ligand is shown for clarity of the allosteric site location. The results are shown for the POPC simulations, and the others can be found in Figure S2. The lipid atoms were selected at a distance of 6 Å from the selected receptor atoms.

The ligand NDT9513727 (IC_50_ = 11.6 nM) sits in a cavity located between helices 3, 4, and 5 in the middle of the lipid bilayer^19,20^ (Figure 2A,B). We simulated the two available NDT9513727-C5aR crystal complexes (PDB ID: 5O9H, chain A and 6C1Q) (Table 1). The receptor is stable with similar dynamics in the simulations of both structures. In contrast, while the ligand from 6C1Q was stable, it had a high fluctuation in the POPC and POPC-Chol and was unstable in the DMPC simulations in the complex from 5O9H (RMSF values in Table 1). The high fluctuation of the ligand is due to one of the benzodioxoles, which relocates from its initial position between helices 3 and 4 into the lipid bilayer. A close examination of the structures has revealed that the side chain of T129^3.45^ has a different orientation in the two X-ray structures (Figure S3). The OH group of T129^3.45^ is pointed toward methylene of benzodioxole in 6C1Q, whereas the CH_3_ group of T129^3.45^ faces methylene in 5O9H. Because the ligand is more stable in 6C1Q, the OH group of T129^3.45^ likely stabilizes the ligand via polar interactions.

We, next, compare the ligand–residue pairwise interaction energy to further explore polar interactions (Figure 2B and Tables S9 and S10). The strongest electrostatic contact of the ligand with an energy of −15.9 kcal/mol in POPC is with W213^5.49^ (Figure 2C). In particular, the imidazole group forms a hydrogen bond with the side chain of W213^5.49^. The hydrogen bond is present in all of the trajectories, with an occupancy of ∼90% of the total frames (Table S2). Mutation of this residue to leucine abolishes the binding of the ligand.^19^ In addition, we see notable electrostatics, in the range of [−2.3, −1.0] kcal/mol with L209^5.45^, A128^3.44^, T217^5.53^, L125^3.41^, A156^4.45^, and T129^3.45^. Among them, the L209^5.45^ backbone is close to one benzodioxole, whereas the A156^4.45^ and L125^3.41^ backbone and the T129^3.45^ side chain are close to another benzodioxole of NDT9513727 (Figure 2C). The importance of T129^3.45^ in the binding of NDT9513727 is confirmed by mutagenesis.^19^ Interestingly, avacopan, another AM crystallized with the receptor forms classical hydrogen bonds with T129^3.45^ and T217^5.53^.^20^ In addition, NDT9513727 forms vdW interactions with W213^5.49^, L125^3.41^, L209^5.45^, L163^4.52^, P214^5.50^, T129^3.45^, and V159^4.48^ with the vdW energy below −2 kcal/mol. NDT9513727 is engaged in weak vdW interactions with three lipid tails in the three membrane systems (Table S9).

In the F/I-SAPT calculations, the electrostatic component of −19.94 kcal/mol between the side chain of W213^5.49^ and the ligand confirms a strong H-bond (Figure 2C and Table S11). Similar to PAR2, the F/I-SAPT calculations highlighted the electrostatic interactions of the backbone amide of L209^5.45^ and A156^4.45^ with values of −5.05 and −4.27 kcal/mol, respectively. The amides of both residues form polar interactions with one of the benzodioxoles at a close distance to form a O–HC bond between the carbonyl of the amide and the methylene hydrogen of the benzodioxole. The electrostatic contribution of the T129^3.45^ and T217^5.53^ side chains was −3.63 and −2.58 kcal/mol, respectively. The OH group of T129^3.45^ is at a distance to form the O–CH bond with one of the benzodioxoles, whereas the OH group of T217^5.53^ is at the closest distance with the aromatic hydrogen of the phenyl ring. Together, the electrostatic interactions with these four residues account for 78% of the electrostatic contribution of W213^5.49^. This shows the notable contribution of weak polar interactions to ligand stability.

The efficiency of the L209^5.45^ and A156^4.45^ amide (−0.99 and −1.11 kcal/mol) and the T129^3.45^ and T217^5.53^ side chain (−1.0 and −0.73 kcal/mol) was about half less than of the W213^5.49^ side chain (−1.64 kcal/mol) (Table S11).

*Q*_CT_ of the ^W213^N(H–N^sp2^) contact is about the reference value, confirming the strong hydrogen bond with the optimal geometry (Figures 2D and S1 and Table S13). In the case of other polar contacts capable to form nonclassical hydrogen bonds, *Q*_CT_ is more than twice smaller of a reference value, indicating very weak interactions. Thus, the attractive polar interactions observed in the F/I-SAPT calculations are mostly due to long-range electrostatics.

In the absence of the allosteric ligand, the fluctuations of the receptor and the allosteric site are in the same range as those observed in the ligand-bound simulations in all of the membrane compositions (Table 1). W213^5.49^ does not form any H-bond with the surrounding amino acids, and on rare occasions, it interacts with water molecules that reach the allosteric binding site from the receptor intrahelical cavity. The allosteric site forms vdW interactions with six lipid tails (Table S6). We see the tips of three lipid tails from the upper and low leaflets occupy the allosteric site in the simulations. The low-energy lipid area in the allosteric site is shown in Figure 2E. Water molecules occupy the pockets of T129^3.45^, A156^4.45^, V159^4.48^, T217^5.53^, and L209^5.45^, forming hydrogen bonds with the occupancy of 28, 12, and 20% in POPC (Figure 2F and Table S7).

According to Fpocket, the allosteric protein cavity is not found to be druggable in the POPC and DMPC simulations, but it is druggable when the lipids are accounted as a part of the cavity. Like in PAR2, the volume of the allosteric cavity is reduced by ∼67% in the absence of the lipids (Figure 2G). The protein cavity volume variation is minimal between the bound and unbound receptor (Table S8). The polar and nonpolar surface area ratio of the protein cavity is 20 and 80%, respectively.

### GCGR in Complex with MK-0893

**Figure 3:**
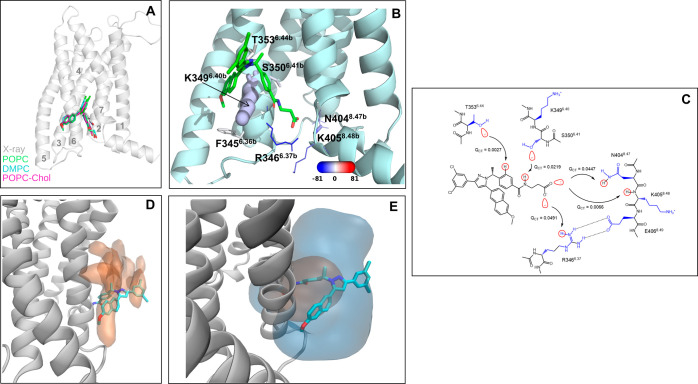
Extrahelical allosteric site of GCGR and its interactions from MD simulations and quantum chemical calculations. (A) Overall location of MK-0893 in GCGR. The overlay of the X-ray position with the average position of MK-0893 in the POPC, DMPC, and POPC-Chol simulations. The helices are labeled. (B) Zoomed view of the binding site. The key residues forming contacts with MK-0893 are shown in stick representation. The size and color of the residues correspond to the relative strength of van der Waals and electrostatic interactions with the ligand, respectively. The actual values of the interaction energies are shown in Table S14. (C) 2D view of the key MK-0893–GCGR interactions and the value of the *Q*_CT_ descriptor. The orbital interactions responsible for charge transfer (*Q*_CT_) between a donor and an acceptor are visualized in red. The direction of charge transfer is shown by arrows. The backbone and the side chain of residues are colored black and blue. (D) Low-energy lipid area (in orange surface) in the allosteric site obtained from the grid free energy calculation based on the POPC simulations of the receptor empty form. (E) Overlay of the allosteric cavities from MDpocket calculation with the selection of only receptor atoms (orange surface) and receptor–lipid atoms (blue surface). The ligand is shown for clarity of the allosteric site location. The results are shown for the POPC simulations, and the others can be found in Figure S2. The lipid atoms were selected at a distance of 6 Å from the selected receptor atoms.

MK-0893 (*K*_d_ = 0.97 nM) is an amphipathic compound that binds on the intracellular side of helices 6 and 7, outside of the helical bundle^23^ (Figure 3A,B). The RMSD and RMSF values (Table 1) show that MK-0893 and the receptor are stable with only small fluctuations in the all simulated membrane compositions. The most dynamic part of the ligand is the 1,3-dichlorobenzene moiety, which is fully exposed to the lipid side.

Unlike the two above ligands, the hydrophilic atoms of MK-0893 have strong electrostatic interactions with several residues: R346^6.37b^, K405^8.48b^, N404^8.47b^, K349^6.40b^, and S350^6.41b^ with the electrostatic energy in the range of [−80.1, −10.6] kcal/mol (Figure 3B and Table S14). In our simulations, we see stable and durable interactions of the ligand carboxyl group with R346^6.37b^ and N404^8.47b^ with an occupancy of above 97 and 80%, respectively, in all of the simulations (Table S2). R346^6.37b^ and N404^8.47b^ are stabilized by direct interactions with E406^8.49^ in helix 8, which further supports the hydrogen bond network (Figure 3B). The hydrogen bond with the backbone of K405^8.48b^ is maintained 10% of the simulation time, whereas the hydrogen bonds with the side chains of K349^6.40b^ or S350^6.41b^ occurred 2% of the time. Our results are in the agreement with the mutagenesis data that show the importance of R346^6.37b^, N404^8.47b^, and K405^8.48b^ and, to a lesser extent, S350^6.41b^ for ligand binding.^23^ In addition, the ligand forms weak vdW interactions with F345^6.36b^, T353^6.44b^, and A348^6.39b^. The ligand has vdW contacts with five lipid tails and a polar interaction with one lipid head group in all of the simulations (Table S14).

The F/I-SAPT calculations confirm strong electrostatic contribution for the side chain of K349^6.40b^, R346^6.37b^, K405^8.48b^, and N404^8.47b^ with the values in the range of [−98.41, −27.5] kcal/mol and weaker electrostatic interactions for the amide of N404^8.47b^ and K405^8.48b^ and the side chain of S350^6.41b^ (Table S15) in the range of [−14.86, −1.02] kcal/mol. The natural population analysis found four classical hydrogen bonds: two strong hydrogen bonds formed by the carboxylate group of the ligand with ^R346^N(H^+^**–**O**^–^**) and ^N404^N(H–O^**–**^) and two weaker interactions with ^S350^H(O–H)N and ^K405^N(H–O**^–^**) (Figure 3C and Table S16).

Unlike the PAR2 and C5aR1 allosteric sites in the middle of the membrane, the GCGR allosteric site is at the border of the water–lipid phase and faces positively and negatively charged lipid head groups, so the electrostatic screening weakens the interactions between the charged groups, explaining the ligand tolerability to R346^6.37b^A mutation.^23^

From MD analysis and F/I-SAPT calculations, K349^6.40b^ has high vdW and electrostatic interaction energies (Figure 3B and Tables S14 and S15), which are attributed to the cation−π interactions with the naphthalene and phenyl moieties of the ligand. The ligand covers this residue forming close contact with the entire side chain. Indeed, the K349^6.40b^ side chain’s efficiency is the highest, −16.8 kcal/mol, among other F/I-SAPT fragments. Mutation of this residue to alanine or methionine significantly reduced the binding of MK-0893.^23^

After removing the ligand, large movements are observed in the intracellular region of helix 5, along the whole helices 6 and 7, and in the overall extracellular side of GCGR in all of the membrane compositions. It has been suggested that the mechanism of the MK-0893 action would be to lock helix 6 in an inactive state, thus the ample motions observed in its absence could agree well with this hypothesis.^23^ The polar residues in the interaction network at helices 7 and 8 reorient to form new interactions with the surrounding residues, lipid head groups, and water molecules. R346^6.37b^ interacts with either S350^6.41b^ or moves away from the helical bundle interacting with lipids and water molecules, whereas N404^7.61b^ is in contact with Y400^7.57b^ with the occupancy of above 60%. We see around four head groups of lipids interacting with the allosteric site, and one lipid occupies the cavity (Table S6). The low-energy lipid area in the allosteric site obtained is shown in Figure 3D.

In all of the simulations performed, the allosteric receptor cavity is detectable but not druggable. Like in C5aR1, the allosteric cavity becomes druggable only when the lipids are included in MDpocket calculations. The allosteric receptor cavity volume variation is equal in the ligand-bound and unbound receptors (Table S8). Similarly to other receptors, we see a loss of volume of ∼69% in the absence of the lipids (Figure 3E). The polar and nonpolar surface area ratios of the allosteric cavity are 39 and 61%, respectively.

## Discussion

We performed MD simulations and quantum chemical calculations to quantify the strength of polar intermolecular interactions in the allosteric sites at the receptor–lipid interface, further characterizing the atom contacts observed in the X-ray structures. The application of two computational techniques with different theoretical principles allowed us to quantify the importance of weak polar interactions. The benefit of such a joint approach was apparent for the AZ3451–PAR2 complex with the allosteric site located in the middle of the membrane. The F/I-SAPT calculations highlighted electrostatic interactions for more residues, particularly the backbone amides, than the force field-based energy calculations. This approach also indicated a weak polar contribution of the O–Br halogen bond. The single classical N–HO hydrogen bond identified in the X-ray structure was weak, and the ligand stabilized its binding through weak polar interactions involving nonclassical O–HC and O–Br bonds, the strength of which is enhanced in such a hydrophobic environment. The F/I-SAPT calculations estimated the electrostatics of these interactions as 170% of the electrostatics of the available classical hydrogen bond. Together these weak polar interactions stabilize the ligand at the receptor–lipid interface, providing a good binding affinity for AZ3451. Because many allosteric molecules were identified via random compound screening and structure–activity relationship of such ligands is narrowed or not available, such quantification of interactions could provide insight into the direction of compound optimization.

In the case of the NDT9513727–C5aR1 complex with the allosteric site also at the middle of the membrane, the single available classical N–HN hydrogen bond represents an anchoring point in the ligand stabilization. NDT9513727 contains two well-polarized benzodioxole groups, which form weak polar interactions with the electrostatics accounting for 78% of the N–HN bond electrostatic contribution. The most traditional case was the MK-0893–GCGR complex, with several strong polar intermolecular interactions that drive the ligand binding. Indeed, the proximity of the allosteric site in GCGR to the solvent makes it possible to establish multiple interactions with polar and charged residues of the receptor.

Although several strategies have been proposed to make a compound to reach the protein–lipid interface,^52^ understanding of the reason why certain ligands are tightly bound at the interface is still being refined with a growing number of experimental ligand–protein complexes. The driving force to form the ligand–protein complex solvated by lipids is distinct from that solvated by water. In water, the polar interactions between a ligand and a protein are in competition with the interactions with water molecules, and to attain good affinity, the obvious step is to exploit nonpolar interactions and add lipophilicity during the ligand optimization. Likewise, but in the opposite way, nonpolar ligand–protein interactions in lipids can be displaced by lipid tails, and thus, polar interactions drive the formation of the ligand–receptor complex. In this case, the weak polar interactions we see in the PAR2 and C5aR1 complexes can provide a significant contribution to the ligand stability. These weak polar interactions cannot be compensated by lipids and therefore, they are energetically preferable. Several weak hydrogen bonds could account for one strong interaction. For example, the magnitude of the O–HC interaction is about one-half of the strength of an O/N–HO/N hydrogen bond.^53^ In addition, in such a hydrophobic environment, the polar interactions become stronger. Gao et al. showed that hydrogen bonds can be up to 1.2 kcal/mol stronger in a hydrophobic environment.^54^ It has been suggested that halogen bond stabilization energy is comparable to a strong hydrogen bond of 5.8 kcal/mol.^55^

Although membrane proteins do not tend to expose polar groups to the lipid interface, the oxygen atom of a backbone amide can be an acceptor of a hydrogen bond, as we see in PAR2 and C5aR1. A hydrogen bond with the backbone carbonyl group of the P2Y1, CB1, and A1 receptors is also formed by an allosteric modulator in the middle of the membrane.^13,14,24^ In this case, a ligand must bear a donor of the hydrogen bond. However, several classical hydrogen bond donors in a ligand can cause difficulties in membrane permeability, therefore, nonclassical hydrogen bond donors such as HC and halogen atoms can be both energetically favorable and with improved membrane permeability.

The receptor allosteric cavities were open in the simulations of the receptor–ligand bound and unbound forms in all simulated lipid compositions. Among the three receptors, the allosteric cavity composed of the PAR2 receptor atoms was only found druggable. The selection of receptor atoms together with the lipid atoms at a distance of 6 Å allowed us to see all of the allosteric sites druggable in the ligand-bound form of the receptor. This is because the lipids notably increased the volume of the cavity. It appears that the allosteric sites investigated here pass a polar surface druggable criteria of 20–40%^56^ but are shortened on the volume size. In the receptor empty forms, the lipid tails fully or partially fill the allosteric cavity in all of the simulated lipid compositions. Although we did not see a substantial difference in the geometry of the allosteric sites between our MD simulations in the three lipid compositions, we anticipate that various lipid compositions likely could vary the size and shape of the allosteric cavity. Therefore, MD simulations of compound hit-receptor complexes from docking can be beneficial in assessing compound stability in the binding site and the role of the lipids. In the case of ligand stability, the ligand located in the middle of the membrane in the AZ3451–PAR2 and NDT9513727–C5aR1 complexes had reduced stability in DMPC compared to POPC and POPC-Chol.

From MD simulations of the receptor empty forms, we often observe water molecules in the allosteric site of all three receptors. Access to the sites is facilitated by the polarity of the intrahelical receptor cavity. Stabilization of water in the sites is provided by interactions with accessible backbone carbonyl oxygen atoms or the side chain of a few polar residues available at the lipid interface. Thus, in blind allosteric site search, if a cavity contains water molecules in simulations, there are possibilities for the cavity to form polar intermolecular contacts and, therefore, such a cavity should be prioritized among others for ligand search.

The provided insights can be useful for screening library generation aiming to explore a chemical space of ligand binding at the protein–lipid interface. A compound library enriched by fragments carrying weak donors could be considered to improve both binding and membrane permeability.

## Methods

### Molecular Dynamics Simulations

The initial structures for PAR2 (PDB code: 5NDZ and 5NDD), C5aR1 (6C1Q and 5O9H), and GCGR (5EE7) in complex with NAMs were obtained from the Protein Data Bank (PDB). Some of the X-ray structures were obtained using the thermostabilizing technology StaR,^57^ so we reverted the amino acid substitutions in the PDB files to the wild-type sequence and modeled the structural regions not resolved in the X-ray structure using Modeller 9v20.^58,59^ The parameters for three NAMs (AZ3451, NDT9513727, and MK-0893) were obtained using the general Amber force field with the AM1BCC charges^60^ and the program Antechamber^61,62^ from AmberTools v18.^63^ The membrane-receptor systems were built in the CHARMM-GUI server^64^ with three different lipid compositions: POPC, DMPC, and POPC + 10% cholesterol. All of the systems have 340 lipids, NaCl 0.15 mM, and a 19 Å solvent layer over each lipid monolayer, up to a total of 120,000 to 140,000 atoms. The CHARMM-GUI files were converted into the Amber format with the programs reduce, pdb4amber, and charmmlipid2amber. All of the simulations were run in Amber 16 and 18^65−67^ with the ff14SB force field^68^ for proteins. The Lipid14 force field^69^ and TIP3P model^70^ were used to parameterize lipid and water molecules, respectively.

The initial energy minimization stage used 5000 steepest descent steps followed by 5000 steps using conjugated gradients. Heating to 310 K was carried out in the NVT ensemble for a total of 25 ps. The Langevin thermostat with a friction coefficient of 1.0 ps^–1^ was used for equilibration and production runs. The equilibration phase with pressure control and a 1 fs timestep was divided into five consecutive steps in which positional restraints in lipid phosphate atoms, waters and ions, and the protein and ligand were released sequentially. A short 50 ns simulation (NPT, 2 fs timestep) was run as the last stage of the equilibration process and discarded from the analysis. Both equilibration and production were run in the NPT ensemble with semi-isotropic pressure control using the Monte Carlo barostat.^71^ The nonbonded force cutoff was set at 10 Å for both van der Waals and electrostatic interactions. The electrostatic interactions were treated with the particle mesh Ewald (PME) method.^72^ Frames were saved every 100 ps to a total of 5000 frames for the initial trajectories (500 ns) and 1500 frames for each of the five replicas (150 ns). In fact, given that we did not see much change in the ligand binding site after 100 ns in the first long 500 ns trajectory, we chose to run short replicas to evaluate the statistical significance of ligand binding interactions. Different production runs were started from the same equilibrated structure. Neither initial velocities nor coordinates were changed, and only different seeds were used for the Langevin thermostat. All of the generated replicas were used for the trajectory analyses.

### Trajectory Analysis

RMSD, RMSF, and hydrogen bond occupancy analyses of the trajectories were performed with VMD 1.9.3.^73^ The residue–ligand interaction energy was calculated using the “namdenergy.tcl” script v1.6 of NAMD.^74^ The residues at a 5 Å distance from the ligand were selected for the interaction energy analysis. Modeling pictures were created with Pymol 2.5.0^75^ and VMD 1.9.3. The structural features and druggable parameters for the allosteric binding sites were analyzed with the program MDpocket,^50^ which allowed assessing the dynamics of the pockets along a trajectory by applying Fpocket 3.0 criteria to detect cavities and assess their druggability.^51^ The grid identified around the allosteric site was used to calculate the volume of the cavity. The SASA of allosteric binding sites, hydrogen bond, and water occupancy were calculated using VMD 1.9.3.^73^ The following residues were selected for SASA calculation: **PAR2**: N116, L119, A120, D121, L123, S124, G153, F154, F155, Y156, G157, N158, M159, C161, F165, I198, W199, L200, I202, L203, L204, T206, I207, Y210, V211; **C5R1**: Y121, I124, L125, L126, L127, A128, T129, I130, S131, A132, I155, A156, C157, A158, V159, A160, W161, L163, L167, V208, L209, G210, F211, W213, P214, L215, T217, L218, F252; **GCGR**: F322, I325, V326, L329, L333, M338, Y343, K344, F345, R346, L347, A348, K349, S350, T351, L352, T353, L354, P356, L395, V398, L399, Y400, C401, F402, L403, N404, K405, E406, V407, and Q408. For the water occupancy, the hydrogen bonds of water molecules with F154, A120, and G157 in PAR2 and T129, A156, V159, L209, and T217 in C5aR1 were calculated. The lipid occupancy analysis was performed using the MDAnalysis package.^76,77^ The occupancy grid was converted into the grid free energy (GFE) by formula 1 based on the ratio between the lipid carbon occupancy around the allosteric site and the average lipid carbon occupancy in the membrane. *R* is the ideal gas constant and *T* is the simulation temperature. The formula was adapted from the grid free energy calculations in SILCS simulations.^78^ The function “min” in formula 1 is used to avoid infinite values of free energy in the protein medium. GFE_max_ is the maximum free energy in a grid cell for this data set of the simulation frames, which was set to 3 kcal/mol to visualize the distribution of lipids around the ligand. The occupancy value was selected as an average lipid carbon occupancy at grid points, which lies at least 10 Å from the protein. The lipid-free energies were taken as an average number from all replicas. Isovalues of −1.2 kcal/mol were used for grid visualization in the figures. The limitations of this approach are the assumption that the values in each grid cell are independent of each other and that one initial lipid configuration is used for the calculations.
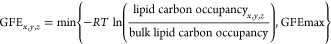
1

### Quantum Chemical Calculation

To perform SAPT calculations, a ligand with surrounding protein residues was cut and capped with acetyl and *N*-methyl terminal groups. The residues involving Y121–T129, A156–A164, and V208–T217 in C5aR1 (6C1Q); M338–L354 and L395–E406 in GCGR; and N116–S124, N149–F165, and S195–V212 in PAR2 were selected for the SAPT calculations.^43^ All of the key residues highlighted by the MD ligand–protein interaction energy decomposition were selected for the SAPT calculations. The overall number of atoms in SAPT was 600–700. The residues without direct contacts with the ligand were mutated to alanine to reduce computational time: S209A, C243A, W247A, L298A, L301A in C5aR1; L122A, L151A, F155A, Y156A, M159A, I163A, L196A, L200A, L204A, and V212A in PAR2; H339A, H340A, W341A, C401A, and F402A in GCGR. Hydrogen atoms were added using Maestro 2021-1, and the geometries of the ligand–receptor complexes were optimized with ORCA 4.2.1.^79^ The optimization process consisted of two steps: first, we constrained all nonhydrogen atoms and optimized hydrogen atoms at the PM3 level and next, the entire structures were optimized at the PBE0 DFT level^80^ with the def2-SVP basis set^81^ and the TIGHTSCF convergence criterion with D3 dispersion correction^82^ using Becke–Johnson damping.^83^ During the last stage, the Cα atom positions were constrained to prevent global structure deformation caused by changes made in the receptors. When the maximal value and root mean square of the energy gradient are below 0.0003 and 0.0001, respectively, the energy minimization is considered to be converged, and the optimization of the complexes is stopped. The obtained coordinates were used for further evaluation. The backbone and the side chain of the residues involved in the allosteric site were selected and capped with hydrogens to perform the F/I-SAPT calculation using psi4 1.3.2.^84^ The natural population analysis with *Q*_CT_ calculation was performed using the JANPA program^45,46^ based on the DFT-level electron density above.

## Notes

The input and output coordinate files are available at this link: DOI 10.5281/zenodo.6684093. ChemDraw 21.00 for 2D diagrams constructed (https://perkinelmerinformatics.com/products/research/chemdraw) is distributed under license. Modeller 9v20 (https://salilab.org/modeller/), AmberTools and Amber16, 18 (https://ambermd.org), and ORCA 4.2.1 (https://www.faccts.de/) packages are available under license. VMD 1.9.3 (https://www.ks.uiuc.edu/Research/vmd/), MDAnalysis (https://www.mdanalysis.org/), and MDpocket and Fpocket (https://github.com/Discngine/fpocket) are open-source codes. The open-source version of Pymol (https://github.com/schrodinger/pymol-open-source) is available free of charge. NAMD (https://www.ks.uiuc.edu/Research/namd/) is available free of charge for academic users. Psi4 (https://github.com/psi4/psi4) is an open-source code. The JANPA (http://janpa.sourceforge.net/) program is an open-source code. Maestro (https://www.schrodinger.com/products/maestro) is a product of Schrödinger, LLC; academic and commercial licenses are available.

## Supporting Information Available

Detailed analysis of MD simulations, F/I-SAPT calculations, and natural population analysis (PDF)
Input files of the equilibration and production protocols of MD simulations (ZIP)
Input files and coordinate files of F/I-SAPT calculations (ZIP)
