# Supplementary material for: Intermolecular Interactions in G Protein-Coupled Receptor Allosteric Sites at the Membrane Interface from Molecular Dynamics Simulations and Quantum Chemical Calculations
Source: J Chem Inf Model. 2022 Sep 30;62(19):4736–47. doi: 10.1021/acs.jcim.2c00788 (PMC9554917; doi:10.1021/acs.jcim.2c00788)
Supplement: Supplementary file 1 — ci2c00788_si_001.pdf [file ci2c00788_si_001.pdf]

Supporting Information

Intermolecular interactions in G protein-coupled receptor allosteric sites at the membrane interface from molecular dynamics simulations and quantum chemical calculations.

Tianyi Ding, Dmitry S. Karlov, Almudena Pino-Angeles and Irina G. Tikhonova

School of Pharmacy, Medical Biology Centre, Queen's University Belfast, Belfast, Northern Ireland, BT9 7BL, UK

* Correspondence, Email: i.tikhonova@qub.ac.uk

Table S1. The average ligand-residue interaction energy, involving van der Waals and electrostatic components in PAR2 from the MD simulations in different lipid compositions.

	POPC			DMPC			POPC + Cholesterol		
**Residue**	E_{vdW}, kcal/mol	E_{ele}, kcal/mol	E_{total}, kcal/mol	E_{vdW}, kcal/mol	E_{ele}, kcal/mol	E_{total}, kcal/mol	E_{vdW}, kcal/mol	E_{ele}, kcal/mol	E_{total}, kcal/mol
**Y210$^{4.61}$**	-4.7	-5.1	-9.5	-3.6	-4.9	-8.5	-5.0	-3.9	-8.9
**F154$^{3.31}$**	-6.4	-1.8	-8.2	-6.7	-1.7	-8.4	-7.0	-2.2	-9.2
**L203$^{4.54}$**	-5.5	-0.8	-6.3	-4.6	0.0	-4.6	-4.8	-0.1	-4.9
**W199$^{4.50}$**	-5.4	-0.6	-6.0	-6.2	-0.8	-7.0	-5.7	-0.7	-6.4
**A120$^{2.49}$**	-1.2	-2.8	-3.9	-1.2	-2.3	-3.5	-1.0	-2.7	-3.8
**G157$^{3.34}$**	-2.2	-1.4	-3.6	-1.7	-0.8	-2.6	-2.1	-1.2	-3.3
**I207$^{4.58}$**	-2.4	-0.8	-3.2	-0.6	-0.2	-0.8	0.0	0.0	0.0
**L123$^{2.52}$**	-3.7	1.0	-2.7	-3.4	0.8	-2.7	-3.7	0.9	-2.7
**T206$^{4.57}$**	-1.4	-0.5	-1.8	-1.2	-0.4	-1.6	-0.8	-0.4	-1.2
**I202$^{4.53}$**	-1.8	0.2	-1.6	-1.9	0.4	-1.5	-1.8	0.5	-1.3
**C161$^{3.38}$**	-0.9	-0.3	-1.2	-0.8	0.1	-0.7	-1.3	-0.2	-1.3
**Lipid tail**	-8.8	0.0	-8.8	-7.9	0.0	-7.9	-5.4	0.0	-5.4
**Lipid tail**	-3.9	-0.1	-4.1	-2.4	0.0	-2.4	-5.0	-0.1	-5.1
**Headgroup**	-1.7	-1.5	-3.2	-0.6	-0.9	-1.5	-	-	-
**Lipid tail**	-1.9	-0.1	-2.0	-1.7	0.0	-1.7	-1.9	0.0	-1.9
**Lipid tail**	-1.1	0.0	-1.1	-1.5	0.0	-1.5	-1.0	0.0	-1.9

Table S2. Hydrogen bond occupancy of the most critical hydrophilic residue in the ligand-bound and unbound receptor in percentage. The threshold for a hydrogen bond was a distance of 3.2 Å and an angle of 120° between donor and acceptor atoms.

System Simulation Runs	PAR2		C5aR1		GCGR			
	Y210 – AZ3451	Y210-Empty	W213-NDT9513727	W213-Empty	R346 – MK-0893	R346–Empty	N404–MK-0893	N404–Empty
**POPC**	65±7	91±3	91±3	42±7	97±4	100	80±4	100
**DMPC**	53±19	99±1	34±1	85±8	100	100	90±1	100
**POPC_Chol**	67±21	63±14	90±0	34±8	100	100	90±8	100
**POPC-5NDD**		88±4						
**POPC_6C1Q**			93					
**DMPC-6C1Q**			90					
**POPC-Chol_6C1Q**			92					

Table S3. The decomposition of the pairwise interaction energy of PAR2 residue backbone amide or sidechain and AZ3451 derived from SAPT0 calculations. N is a number of non-hydrogen atoms in a protein fragment.

**Receptor Fragment**	**Electrostatics, kcal/mol**	**Exchange, kcal/mol**	**Induction L→R*, kcal/mol**	**Induction R→L*, kcal/mol**	**Dispersion, kcal/mol**	**Total, kcal/mol**	**Total / N**, kcal/mol**
Y210-sidechain	-8.58	9.31	-1.62	-1.53	-10.35	-12.77	-1.60
W199-sidechain	-4.3	9.8	-0.1	-0.23	-11.11	-6.85	-0.69
F154-amide-F155	-5.5	2.34	-1.06	-0.32	-1.37	-5.91	-1.97
L203-sidechain	-3.36	9.32	-0.2	-0.27	-9.39	-3.91	-0.98
W199-amide-L200	-3.72	4.29	-0.67	-0.25	-2.54	-2.89	-0.96
A120-amide-D121	-5.37	6.15	-0.57	-0.79	-1.77	-2.35	-0.78
L203-amide-L204	-1.14	0.02	-0.16	-0.02	-0.46	-1.76	-0.59
F154-sidechain	-3.16	8.98	-0.5	-0.36	-6.35	-1.4	-0.2
L119-amide-A120	-0.5	0.04	-0.13	-0.01	-0.28	-0.89	-0.2
L123-sidechain	-2.15	9.8	-0.01	-0.31	-8.08	-0.74	-0.19
C161-sidechain	-3.04	6.69	-0.24	-0.61	-3.49	-0.7	-0.35
T206-sidechain	-0.15	0.01	0.05	-0.03	-0.55	-0.68	-0.23
Y156-amide-G157	-0.15	0.01	-0.13	-0.01	-0.36	-0.64	-0.21
L202-sidechain	-1.33	4.39	-0.18	-0.13	-3.25	-0.5	-0.13
I198-amide-W199	0.16	0.01	-0.29	0	-0.28	-0.4	-0.13
T206-amide-I207	0.1	0.01	-0.24	-0.02	-0.24	-0.39	-0.13
D121-amide-L122	-0.1	0	-0.09	-0.01	-0.06	-0.26	-0.08
I207-sidechain	0.09	2.08	-0.02	-0.22	-2.19	-0.26	-0.07
G153-amide-F154	0.25	0.01	0.19	-0.03	-0.47	-0.05	-0.01
V205-amide-T206	0.16	0	-0.06	0	-0.08	0.02	0.00
C161-amide-S162	0.27	0	0.17	0	-0.07	0.37	0.12
G157-amide-N158	-1.77	6.47	-0.15	-0.23	-3.93	0.41	0.14
L209-amide-Y210	0.52	0	0.07	-0.01	-0.11	0.47	0.15
A120-sidechain	0.71	0.45	0.11	-0.04	-0.74	0.48	0.48
Y160-amide-C161	0.43	0	0.2	-0.01	-0.14	0.5	0.16
Y210-amide-V211	0.67	0	0.04	-0.01	-0.19	0.5	0.16
I207-amide-sidechainPro208	1.02	0.02	-0.07	-0.02	-0.32	0.62	0.09
L123-amide-S124	0.83	0.02	0.21	-0.03	-0.33	0.7	0.23
L122-amide-L123	0.75	0	0.16	-0.01	-0.13	0.77	0.25
L202-amide-L203	-0.09	3.1	0.22	-0.15	-2.07	1.01	0.34

*L→R/ R→L: Ligand→Receptor/Receptor→Ligand; **Total/N is fragment efficiency

Table S4. The decomposition of the pairwise interaction energy between the PAR2 receptor fragments and the AZ3451 amidobenzonitrile (ABN), benzimidazole (BI), or bromobenzodioxol (BBDO) fragments derived from SAPT0 calculations.

Receptor Fragment	Ligand Fragment	Electrostatics, kcal/mol	Exchange, kcal/mol	Induction L→R, kcal/mol	Induction R→L, kcal/mol	Dispersion, kcal/mol	Total, kcal/mol
Y210-sidechain	ABN	-1.94	4.19	-0.56	-1.13	-5.50	-4.94
Y210-sidechain	BI	-9.25	9.49	-1.49	-0.91	-5.47	-7.62
F154-amide-F155	BBDO	-4.37	2.34	-1.12	-0.28	-1.39	-4.83
A120-amide-D121	BBDO	-4.45	6.15	-0.54	-0.78	-1.84	-1.46

Table S5. Q_{CT} devised from natural population analysis for atom-pairwise contacts in the AZ3451-PAR2 complex, the distance and angle of these contacts from the X-ray and optimized structures.

Two atom contact (receptor-ligand)	Interacting orbitals	Q_{CT}, a.u.	Optimized distance, Å	Optimized angle, ° (expected from VSEPR*)	X-ray distance, Å	X-ray angle, °	VdW sum, Å
^{A120b}(**O-H**)C^{sp3}	n(O) -> σ*(H-C)	0.0100	2.12	115 (120)	2.44	96	2.72
^{F154b}(**O-H**)C^{sp3}	n(O) -> σ*(H-C)	0.0024	2.38	131 (120)	3.05	133	2.72
^{W199b}(**O-Br**)	n(O) -> σ*(Br-C)	0.0032	3.08	135 (120)	2.97	145	3.05
^{Y210}O(**H-N**^{sp2})	n(N) -> σ*(H-O)	0.0128	2.25	151 (120)	2.91	150	2.75
^{F154}C(**H-O**^{sp2})	n(O) -> σ*(H-C)	0.0053	2.13	131 (104)	2.35	91	2.72

*VSEPR – Valence shell electron pair repulsion (VSEPR) theory

n(O/N) is the orbital corresponding to a lone pair of electrons. σ*(H-C/H-O/Br-C) is a non-bonding orbital.

Q_{CT} is charge transfer from the occupied orbital with a lone pair of electrons to the non-bonding orbital.

Table S6. The average allosteric site-lipid interaction energy, involving van der Waals and electrostatic components in kcal/mol from the MD simulations of the receptor empty form.

Receptor	Lipid	POPC			DMPC			POPC + Cholesterol		
		$E_{vdW,}$	E_{ele}	E_{total}	E_{vdW}	E_{ele}	E_{total}	E_{vdW}	E_{ele}	E_{total}
**PAR2**	**Headgroup**	-0.4	-2.4	-2.9	-2.7	-8.6	-11.3	0	0	0
	**Lipid tail**	-15.3	-0.6	-15.9	-9.8	-0.1	-10.0	-11.5	0	-11.5
	**Lipid tail**	-6.0	-0.2	-6.2	-1.8	0.1	-1.6	-5.4	-0.2	-5.6
	**Lipid tail**	-3.1	0	-3.2	-0.4	-0.9	-1.4	-1.0	0	-1.0
	**Lipid tail**	-1.4	0	-1.4	0	0	0	-0.3	-0.3	-0.6
**C5aR1**	**Lipid tail**	-5.1	-0.1	-5.2	-15.0	-0.3	-15.2	-7.7	-0.2	-7.9
	**Lipid tail**	-5.3	-0.1	-5.4	-6.5	-0.2	-6.7	-3.8	0.1	-3.9
	**Lipid tail**	-3.9	-0.1	-4.0	-4.9	-0.3	-5.2	-3.7	0.1	-3.8
	**Lipid tail**	-2.3	0	-2.3	-2.4	0	-2.3	-3.5	-0.1	-3.6
	**Lipid tail**	-2.1	0	-2.2	-2.1	0	-2.2	-2.3	-0.1	-2.4
	**Lipid tail**	-1.7	0	-1.7	-1.7	0	-1.7	-1.7	0	-1.7
**GCGR**	**Headgroup**	-2.8	-64.0	-67.0	-3.5	-83.2	-86.6	-1.9	-68.1	-70.0
	**Headgroup**	-2.6	-60.0	-63.0	-8.1	-77.1	-85.1	-0.8	-48.3	-49.0
	**Headgroup**	-1.2	-8.1	-9.3	-0.6	-55.8	-56.4	-0.4	-6.3	-6.7
	**Headgroup**	-0.4	-3.9	-4.3	0	-2.0	-2.0	-0.2	-1.4	-1.6
	**Headgroup**	-0.2	-2.3	-2.5	0	0	0	0	0	0
	**Headgroup**	0	-1.5	-1.5	0	0	0	0	0	0
	**Lipid tail**	-2.4	-0.2	-2.6	-7.0	-0.5	-7.5	-6.0	0.2	-5.8
	**Lipid tail**	0	0	0	-4.4	-0.4	-4.7	-1.6	-0.1	-1.7

Table S7. The average water occupancy in the empty form of PAR2 and C5aR1 allosteric sites from the MD simulations. The threshold for a hydrogen bond was a distance of 3.5 Å and an angle of 135° between donor and acceptor atoms. A120, F154 and G157 were selected in PAR2. In C5aR1 water molecules were monitored in three sites: (1) T129, A156 and V159; (2) T217 and (3) L209.

Systems	% Occupancy
PAR2-E	
POPC	51%±0.1
DMPC	42%±0.2
POPC-Chol	14%±0.2
PAR2_5NND	19%±0.1
C5aR1-E	
POPC (1)	28%±0.3
DMPC (1)	26%±0.1
POPC-Chol (1)	8%±0.1
POPC (2)	12%±0.1
DMPC (2)	59%±0.2
POPC-Chol (2)	0.0%±0.0
POPC (3)	20%±0.1
DMPC (3)	2%±0.2
POPC-Chol (3)	5%±0.01

Table S8. Druggability, volume and solvent accessible surface area (SASA) of allosteric binding sites in PAR2, C5aR1 and GCGR in the ligand-bound (-L) and empty forms (-E) in three membrane compositions. Druggability and volume of the cavities were assessed by the MD-pocket program using the Fpocket druggability criteria. The cavities were selected based on the selection of only the receptor residues shown in the Methods[1] or the receptor residues together with lipid atoms at the distance of 6 Å from these residues[2]. $SASA_{phil}$, and $SASA_{phob}$ are solvent-accessible surface area for the hydrophilic and hydrophobic residues.

Systems	Pocket type[1]	Pocket type[2]	Volume[1], $Å^3$	Volume[2], $Å^3$	$SASA_{phil}$, $Å^2$	$SASA_{phob}$, $Å^2$
**PAR2**						
POPC-L	Druggable	Druggable	361 ± 52	893±134	233±16	1031±34
POPC-E	Druggable	No pocket	519 ± 107	No pocket	215±28	998±54
DMPC-L	Druggable	Druggable	341 ± 51	913±153	228±23	956±38
DMPC-E	Druggable	No pocket	404 ± 75	No pocket	237±30	986±42
POPC_Chol-L	Druggable	Druggable	354 ± 47	792±205	226±21	994±37
POPC_Chol-E	Druggable	No pocket	281 ± 85	No pocket	187±23	975±50
**C5aR1**						
POPC-5O9H_L	Non-druggable	Druggable	300 ± 92	809±178	250±20	978±29
POPC-5O9H_E	Non-druggable	No pocket	218 ± 103	No pocket	259±28	1010±45
DMPC-5O9H_L	Non-druggable	Druggable	275 ± 119	970±136	261±22	989±38
DMPC-5O9H_E	Non-druggable	No pocket	417 ± 118	No pocket	273±21	1025±48
POPC_Chol-5O9H_L	Druggable	Druggable	238 ± 72	819±152	281±22	1123±66
POPC_Chol-5O9H_E	Non-druggable	No pocket	197 ± 112	No pocket	256±28	968±49
POPC_6C1Q-L	Non-druggable	Druggable	362±84	885±194	170±16	1523±78
DMPC_6C1Q-L	Non-druggable	Druggable	349±94	1124±186	195±18	1618±88
POPC_Chol_6C1Q-L	Non-druggable	Druggable	326±87	953±180	202±16	1495±98
**GCGR**						
POPC-L	Non-druggable	Druggable	374 ± 82	1442±245	415±42	670±43
POPC-E	Non-druggable	No pocket	350 ± 141	No pocket	558±102	772±59
DMPC-L	Non-druggable	Druggable	401 ± 84	1109±170	401±49	701±49
DMPC-E	Non-druggable	No pocket	274 ± 113	No pocket	464±98	784±63
POPC_Chol-L	Non-druggable	Druggable	373 ± 75	1249±219	390±41	715±38
POPC_Chol-E	Non-druggable	No pocket	401 ± 84	No pocket	531±89	686±48

Table S9. The average ligand-residue interaction energy, involving van der Waals and electrostatic components in C5aR1 (PDB ID: 6C1Q) from the MD simulations in different lipid compositions.

	POPC			DMPC			POPC + Cholesterol		
**Residue**	E_{vdW}, kcal/mol	E_{ele}, kcal/mol	E_{total}, kcal/mol	E_{vdW}, kcal/mol	E_{ele}, kcal/mol	E_{total}, kcal/mol	E_{vdW}, kcal/mol	E_{ele}, kcal/mol	E_{total}, kcal/mol
**W213$^{5.49}$**	-5.0	-10.8	-15.9	-5.0	-11.1	-16.1	-4.8	-11.2	-16.0
**L125$^{3.41}$**	-5.4	-1.0	-6.5	-4.9	-1.4	-6.3	-4.9	-1.3	-6.2
**L209$^{5.45}$**	-2.5	-2.3	-4.8	-2.3	-2.3	-4.7	-2.5	-2.3	-4.8
**V159$^{4.48}$**	-2.5	-0.4	-4.8	-2.5	-0.1	-2.6	-2.4	-0.1	-2.5
**T129$^{3.45}$**	-2.1	-1.7	-3.8	-1.2	-1.2	-2.4	-2.1	-1.0	-3.1
**L163$^{4.52}$**	-2.4	0.0	-2.4	-2.2	0.0	-2.2	-2.4	-0.1	-2.5
**T217$^{5.53}$**	-1.1	-1.2	-2.3	-1.0	-1.0	-2.0	-0.9	-1.2	-2.1
**P214$^{5.50}$**	-2.6	0.3	-2.2	-2.6	0.2	-2.4	-2.3	0.2	-2.1
**A156$^{4.45}$**	-1.1	-1.0	-2.1	-1.0	-1.4	-2.4	-1.0	-1.3	-2.3
**A128$^{3.44}$**	0.4	-2.2	-1.8	-2.5	0.4	-2.1	-2.6	0.3	-2.3
**Y121$^{3.37}$**	-0.4	-0.7	-1.1	0	0	0	-0.3	-0.7	1.0
**I124$^{3.40}$**	-1.0	0.3	-0.7	-1.7	0.0	-1.7	-1.7	0.1	-1.6
**Lipid tail**	-2.2	0.0	-2.2	-6.0	-0.1	-6.1	-6.1	-0.1	-6.2
**Lipid tail**	-1.7	0.0	-1.7	-2.2	0.0	-2.2	-2.9	0.0	-3.0
**Lipid tail**	-1.5	0.0	-1.5	-0.6	0.0	-0.6	-1.8	-0.1	-1.9

Table S10. The average ligand-residue interaction energy, involving van der Waals and electrostatic components in C5aR1 (PDB ID: 5O9H) from the MD simulations in different lipid compositions.

	POPC			DMPC			POPC + Cholesterol		
**Residue**	E_{vdW}, kcal/mol	E_{ele}, kcal/mol	E_{total}, kcal/mol	E_{vdW}, kcal/mol	E_{ele}, kcal/mol	E_{total}, kcal/mol	E_{vdW}, kcal/mol	E_{ele}, kcal/mol	E_{total}, kcal/mol
**W213$^{5.49}$**	-5.2	-11.0	-16.2	-3.4	-4.5	-7.9	-5.3	-10.6	-15.9
**L125$^{3.41}$**	-4.6	-0.9	-5.5	-3.9	-1.3	-5.2	-4.2	-0.8	-5.0
**L209$^{5.45}$**	-2.8	-2.2	-5.0	-1.5	-0.7	-2.2	-2.6	-1.9	-4.5
**L163$^{4.52}$**	-2.7	0.2	-2.5	-1.1	0.3	-0.8	-2.3	0.3	-2.0
**T217$^{5.53}$**	-1.1	-1.2	-2.3	-.2.4	-0.2	-2.5	-1.1	-1.0	-2.1
**P214$^{5.50}$**	-2.4	0.3	-2.1	-1.4	0.8	0.6	-2.5	0.2	-2.3
**T129$^{3.45}$**	-1.6	-0.5	-2.1	-4.0	-0.7	-4.7	-1.9	-0.7	-2.5
**A128$^{3.44}$**	-2.2	0.3	-1.9	-3.9	-1.3	-5.2	-2.4	0.2	-2.3
**V159$^{4.48}$**	-1.6	-0.2	-1.8	-2.1	0	-2.1	-2.0	-0.6	-2.6
**I124$^{3.40}$**	-1.5	0.3	-1.2	-0.8	-0.4	-1.2	-1.5	0.1	-1.4
**Y121$^{3.37}$**	-0.4	-0.8	-1.2	0	0	0	0	0	0
**A156$^{4.45}$**	-0.5	-0.6	-1.1	0	0	0	-0.7	-0.2	-1.0
**Lipid tail**	-2.4	0	-2.4	-5.4	0	-5.4	-4.0	0	-4.0
**Lipid tail**	-1.8	0	-1.8	-5.4	0	-5.4	-2.5	0	-2.5
**Lipid tail**	-1.5	0	-1.5	-5.3	0	-5.3	-2.2	0	-2.2
**Lipid tail**	-1.3	0	-1.3	-1	0	-1	-1.8	0	-1.8
**Lipid tail**	-1.0	0	-1.0	-1	0	-1	-1.7	0	-1.7
**Headgroup**	0	0	0	-6.2	-3.5	-9.7	-1.0	0	-1.0

Table S11. The decomposition of the pairwise interaction energy of a C5aR1 residue backbone or sidechain and NDT9513727 derived from SAPT0 calculations. N is a number of non-hydrogen atoms in a protein fragment.

Receptor Fragment	Electrostatics, kcal/mol	Exchange, kcal/mol	Induction L→R*, kcal/mol	Induction R→L*, kcal/mol	Dispersion, kcal/mol	Total, kcal/mol	Total / N**, kcal/mol
W213-sidechain	-19.94	24.75	-5.79	-2.27	-13.12	-16.37	-1.64
A156-amide-C157	-4.27	2.82	-0.33	-0.27	-1.29	-3.33	-1.11
T129-sidechain	-3.63	3.1	-0.12	-0.22	-2.14	-3.02	-1.00
L209-amide-G210	-5.05	4.6	-0.29	-0.19	-2.04	-2.96	-0.99
L125-amide-L126	-1.07	0.18	-0.88	-0.11	-0.01	-2.56	-0.85
L125-sidechain	-1.67	5.83	0	-0.33	-6.28	-2.45	-0.61
T217-sidechain	-2.58	3.72	-1.2	0.19	-1.96	-2.2	-0.73
Pro214-amide-L215	-1.13	0.05	-0.62	-0.01	-0.39	-2.1	-0.70
L209-sidechain	-0.95	2.21	-0.11	-0.14	-2.89	-1.89	-0.47
A128-amide-T129	-0.89	2.36	-1.01	-0.05	-2.12	-1.71	-0.57
I124-amide-L125	-0.65	0.4	-0.55	-0.02	-0.85	-1.68	-0.56
W213-amide-Pro214 + Pro214-sidechain	-2.22	8.48	-1	-0.24	-6.66	-1.63	-0.23
V168-sidechain	-0.51	2.7	-0.05	-0.06	-3.66	-1.48	-0.49
I164-amide-A165	-0.03	0	0.05	0	-0.114	-0.94	-0.31
T217-amide-L218	0.1	0.1	-0.54	-0.02	-0.52	-0.87	-0.29
A169-sidechain	-0.59	0.77	-0.1	-0.08	-0.81	-0.8	-0.8
L212-amide-W213	-0.2	0	-0.23	0	-0.04	-0.47	-0.16
Y121-amide-A122	-0.27	0	-0.07	0	-0.08	-0.41	-0.13
A165-sidechain	0.07	0.06	-0.03	-0.02	-0.36	-0.29	-0.29
M120-amide-Y121	-0.16	0	-0.09	0	-0.02	-0.28	-0.09
Y121-sidechain	0.39	0.14	0.1	-0.03	-0.83	-0.24	-0.03
V208-amide-L209	0	0.03	-0.04	-0.01	-0.2	-0.21	-0.07
A167-amide-V168	0.11	0	0.03	0	-0.11	0.02	0.00
L216-amide-T217	0.09	0	0.22	0	-0.13	0.17	0.06
A169-amide-W170	0.21	0	0.11	0	0.15	0.18	0.06
T129-amide-I130	0.24	0.02	0.32	0	-0.19	0.39	0.13
V168-amide-A169	1.09	0.32	0.21	-0.05	-0.86	0.71	0.24

*L→R/ R→L: Ligand→Receptor/Receptor→Ligand; **Total/N is fragment efficiency

Table S12. The decomposition of the pairwise interaction energy between the C5aR1 receptor fragments and the NDT9513727 and benzdioxole (BDO) fragments derived from SAPT0 calculations.

Receptor Fragment	Ligand Fragment	Electrostatics, kcal/mol	Exchange, kcal/mol	Induction L→R, kcal/mol	Induction R→L, kcal/mol	Dispersion, kcal/mol	Total, kcal/mol
A156-amide-T157	BDO	-3.89	2.82	-0.34	-0.26	-1.30	-2.97
T129-sidechain	BDO	-2.55	1.50	-0.37	-0.133	-1.02	-2.58
L209-amide-G210	BDO	-5.23	4.60	-0.72	-0.19	-0.98	-3.52

Table S13. Q^{CT} devised from natural population analysis for atom-pairwise contacts in the NDT9513727-C5aR1 complex, the distance and angle of these contacts from the X-ray and optimized structures.

Two atom contact (receptor-ligand)	Interacting orbitals	Q^{CT}, a.u.	Optimized distance, Å	Optimized angle, ° (expected from VSEPR**)	X-ray distance, Å	X-ray angle, °	VdW sum, Å
^{A156}(**O-H**)C^{sp3}	n(O) -> σ*(H-C)	0.0031	2.32	129 (120)	2.31	129	2.72
^{T129}H(**O-H**)C^{sp3}	n(O) -> σ*(H-C)	0.0011	2.54	142 (120)	2.36	145	2.72
^{L209}(**O-H**)C^{sp3}	n(O) -> σ*(C-O)	0.0018	2.44	118 (120)	2.52	143	2.72
^{W213}N(**H-N**^{sp2})	n(N) -> σ*(H-N)	0.0572	1.81	121 (120)	1.90	115	2.75
^{T217}H(**O-H**)C^{sp2}	n(O) -> σ*(H-C)	0.0027	2.37	128 (120)	2.06	148	2.72

**VSEPR – Valence shell electron pair repulsion (VSEPR) theory

Table S14. The average ligand-residue interaction energy, involving van der Waals and electrostatic components in GCGR from the MD simulations in different lipid compositions.

	POPC			DMPC			POPC + Cholesterol		
Residue	E_{vdW}, kcal/mol	E_{ele}, kcal/mol	E_{total}, kcal/mol	E_{vdW}, kcal/mol	E_{ele}, kcal/mol	E_{total}, kcal/mol	E_{vdW}, kcal/mol	E_{ele}, kcal/mol	E_{total}, kcal/mol
R346$^{6.37b}$	-1.0	-80.8	-81.8	-0.8	-77.3	-78.2	-69.1	-0.5	-69.5
K405$^{8.48b}$	-0.8	-47.9	-48.7	-0.9	-30.8	-31.7	-16.9	-0.9	-17.8
N404$^{8.47b}$	-0.6	-30.9	-31.5	-0.5	-34.8	-35.4	-37.6	-0.9	-38.5
K349$^{6.40b}$	-10.3	-16.8	-27.1	-10.2	-13.4	-23.6	-11.4	-10.2	-21.6
S350$^{6.41b}$	-2.2	-8.4	-10.6	-2.2	-7.4	-9.7	-8.9	-2.2	-11.2
F345$^{6.36b}$	-1.8	-2.3	-4.0	-1.8	-2.1	-3.9	-1.6	-2.2	-3.8
T353$^{6.44b}$	-2.6	0.4	-2.2	-2.8	0.4	-2.4	0.2	-2.7	-2.5
L347$^{6.38b}$	-0.7	-0.8	-1.5	-1.1	-1.0	-2.1	-0.7	-1.6	-2.2
A348$^{6.39b}$	-1.2	-0.1	-1.3	-1.2	-0.1	-1.3	0.3	-1.3	-1.0
Lipid tail	-7.0	-0.1	-7.1	-8.4	-0.2	-8.6	-8.6	-0.2	-8.8
Lipid tail	-4.8	-0.2	-5.0	-3.6	-0.1	-3.7	-3.6	-0.9	-4.6
Lipid tail	-4.5	-0.2	-4.7	-3.0	0	-3.0	-3.3	-0.1	-3.4
Lipid tail	-2.6	0	-2.7	-1.7	0	-1.7	-2.4	-0.1	-2.5
Lipid tail	-1.7	0	-1.7	-2.2	0.6	-1.7	-1.0	0	-1.0
Headgroup	-1.5	-2.4	-3.9	-2.2	-3.4	-5.6	-1.1	-1.4	-2.5

Table S15. The decomposition of the pairwise interaction energy of a GCGR residue backbone or sidechain and MK-0893 derived from SAPT0 calculations. N is a number of non-hydrogen atoms in a protein fragment.

Receptor Fragment	Electrostatics, kcal/mol	Exchange, kcal/mol	Induction L→R*, kcal/mol	Induction R→L*, kcal/mol	Dispersion, kcal/mol	Total, kcal/mol	Total / N**, kcal/mol
K349-sidechain	-98.41	56.24	-34.98	-2.74	-20.85	-100.73	-16.8
R346-sidechain	-85.83	26.34	-8.35	-9.89	-7.77	-85.5	-12.2
K405-sidechain	-39.75	0.19	-2.75	-1.56	-0.51	-44.38	-7.4
N404-sidechain	-27.5	17.88	-2.06	-5.77	-4.54	-21.9	-5.5
N404-amide-K405	-14.86	1.24	-0.77	-1.78	-1.01	-17.17	-5.7
K405-amide-E406	-7.04	0	-0.17	-0.37	-0.07	-7.64	-2.55
A348-amide-K349	-2.98	0.55	-2.31	0.07	-1.94	-6.74	-2.25
F345-amide-R346	-3.87	0.78	0.27	-0.38	-1.02	-4.22	-1.41
T353-sidechain	-6.12	13.26	-2.569	-0.4	-7.8	-3.63	-1.23
R347-amide-A348	-1.97	0	-1.06	-0.12	-0.24	-3.38	-1.13
S350-amide-T351	-1.02	0.01	-1.7	-0.04	-0.36	-3.1	-1.03
F345-sidechain	-1.95	4.93	0.14	-0.57	-5.2	-2.64	-0.38
S350-sidechain	-8.32	12.79	-0.73	-1.36	-4.39	-2.01	-1.00
L352-amide-T353	-1.55	0.02	0.26	-0.02	-0.49	-1.77	-0.59
A348-sidechain	-0.69	0.7	0.11	-0.03	-1	-0.92	-0.92
L347-sidechain	-0.2	0	0.104	-0.42	-0.16	-0.68	-0.17
A344-amide-F345	0.21	0	-0.34	-0.07	-0.1	-0.3	-0.1
S352-amide-L354	-0.18	0.03	0.16	0	-0.13	-0.12	-0.04
K349-amide-S350	-3.1	8.04	1.76	-0.45	-4.94	1.32	0.44
R346-amide-L347	1.35	1.08	1.62	-0.54	-1.4	2.11	0.70
L403-amide-N404	5.29	6.97	0.79	-0.8	-3.2	9.05	3.02
E406-sidechain	63.59	0.06	3.71	-0.84	-0.16	66.36	13.27

*L→R/ R→L: Ligand→Receptor/Receptor→Ligand; **Total/N is fragment efficiency

Table S16. Q^{CT} devised from natural population analysis for atom-pairwise contacts in the MK-0893-GCGR complex, the distance and angle of these contacts from the X-ray and optimized structures.

Two atoms (receptor-ligand)	Interacting orbitals	Q^{CT}, a.u.	Optimized distance, Å	Optimized angle, ° (expected from VSEPR*)	X-ray distance, Å	X-ray angle, °	VdW sum, Å
$^{R346}N(\mathbf{H^{+}\text{-}O^{-}})$	n(O) -> σ*(H-N)	0.0491	1.79	115 (120)	1.63	149	2.72
$^{N404}N(\mathbf{H\text{-}O^{-}})$	n(O) -> σ*(H-N)	0.0447	1.74	139 (120)	1.73	102	2.72
$^{K405}N(\mathbf{H\text{-}O^{-}})$	n(O) -> σ*(H-N)	0.0066	2.54	106 (120)	2.15	106	2.72
$^{S350}H(\mathbf{O\text{-}H})N$	n(O) -> σ*(H-N)	0.0219	1.91	163 (104)	2.14	157	2.72
$^{T353}H(\mathbf{O\text{-}H})C^{sp2}$	n(O) -> σ*(H-C)	0.0027	2.46	161 (104)	3.11	113	2.72

*VSEPR – Valence shell electron pair repulsion (VSEPR) theory

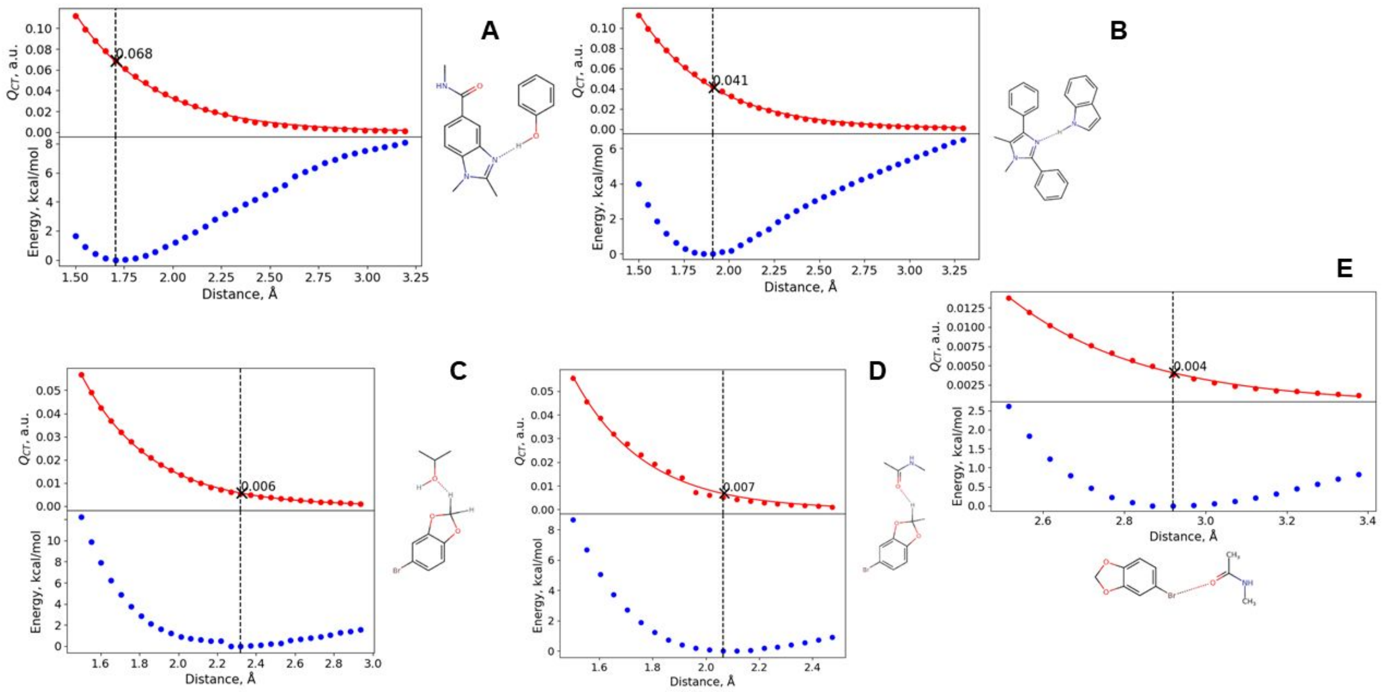

Figure S1. The charge transfer values (Q_{CT}) for optimal geometries of the reference complexes. The distance between interacting atoms was varied to find the optimum geometry while the angle defining the hydrogen bond geometry was constrained as expected from the valence shell electron pair repulsion (VSEPR) theory: 120° was set for C=O---H, C=O---Br, and C=N(-C)---H angles, while 109° was for C-O(-H)---H. Q_{CT} is shown for the optimal geometry.

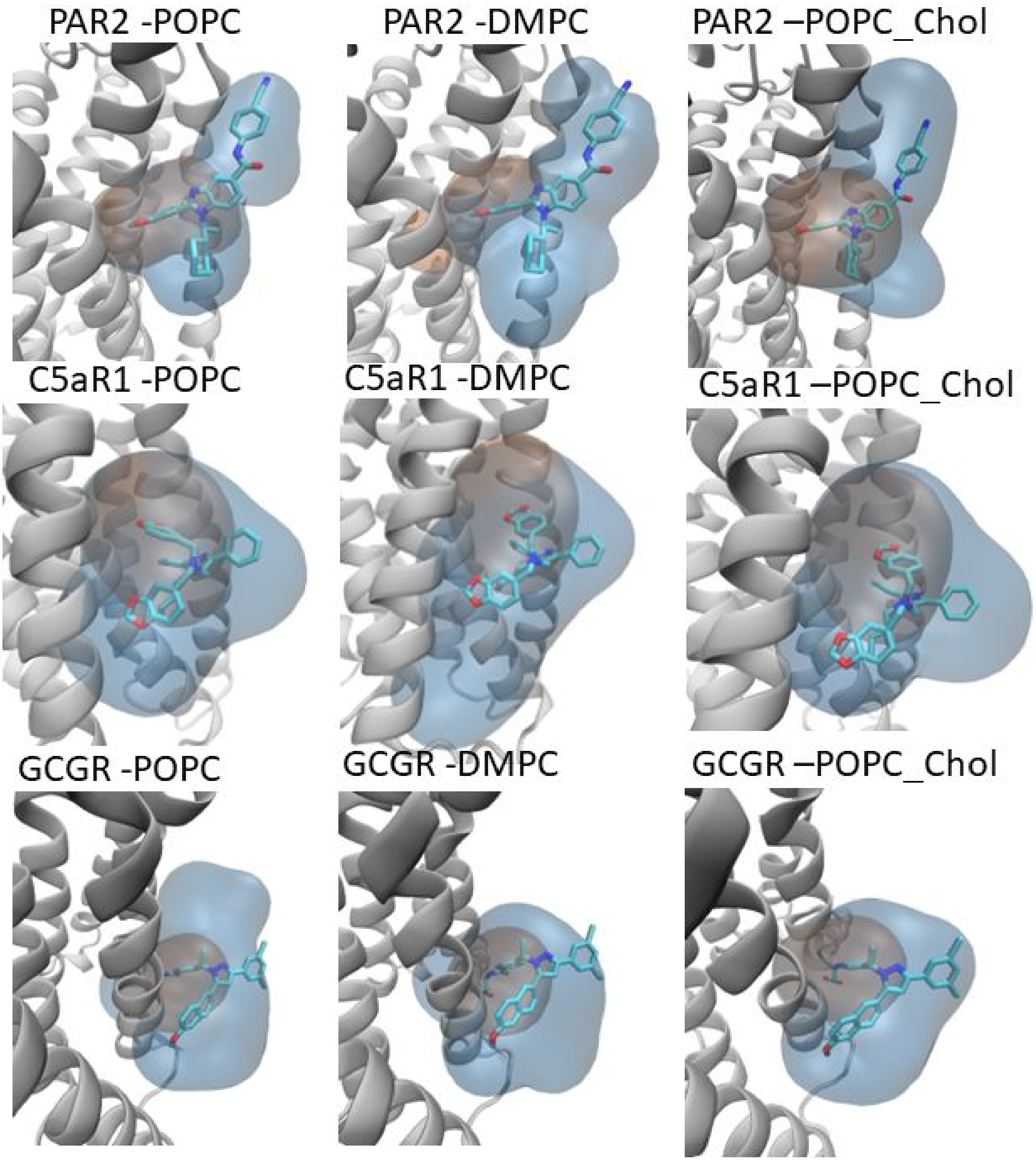

Figure S2. The overlay of the allosteric cavities from MDpocket calculation with selection of only receptor atoms (orange surface) and receptor-lipid atoms (blue surface).

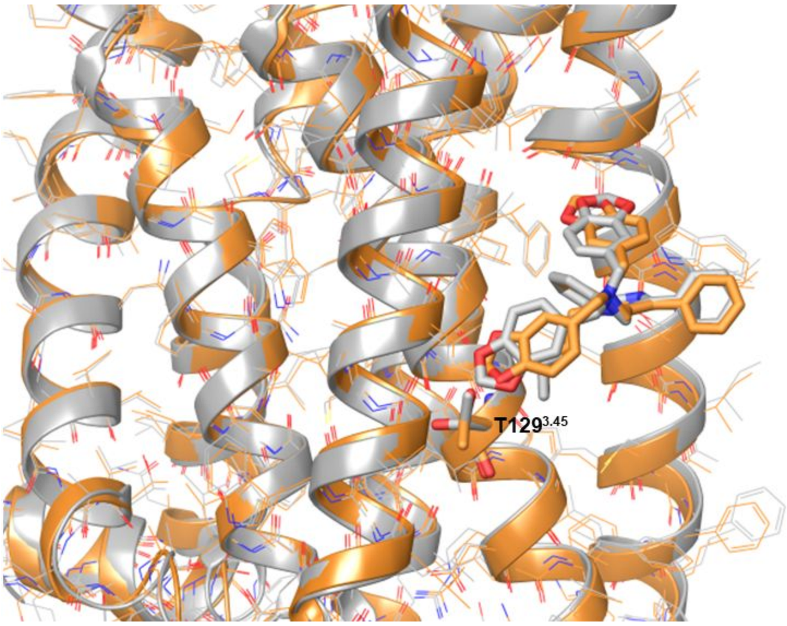

Figure S3. The overlay of the C5aR1 crystal structures with PDB ID 5O9H, Chain A (grey) and 6C1Q (orange).
